# Addition of anterolateral ligament reconstruction to primary anterior cruciate ligament reconstruction could benefit recovery of functional outcomes

**DOI:** 10.1038/s41598-024-62444-x

**Published:** 2024-05-20

**Authors:** Jin Hyuck Lee, Gyu Bin Lee, WooYong Chung, Seung-Beom Han, Ki-Mo Jang

**Affiliations:** 1 Department of Sports Medical Center, Anam Hospital, Korea University College of Medicine, Seoul, 02841 Republic of Korea; 2 Department of Orthopedic Surgery, Anam Hospital, Korea University College of Medicine, Seoul, 02841 Republic of Korea

**Keywords:** Knee, Anterior cruciate ligament reconstruction, Anterolateral ligament reconstruction, Muscle strength, Kinesiophobia, Functional performance, Anatomy, Health care, Prognosis

## Abstract

This study aimed to compare functional outcomes sequentially up to 1 year after combined anterior cruciate ligament reconstruction (ACLR) and anterolateral ligament reconstruction (ALLR) and isolated ACLR. Fifty patients who underwent ACLR with versus without ALLR were analyzed at four different time points (preoperatively and 3, 6, and 12 months postoperatively). For the functional outcomes, muscle strength and acceleration time (AT) were measured using an isokinetic dynamometer. Proprioception was evaluated using joint position sense and dynamic postural stability. Patient-reported outcomes were measured using the Tampa Scale for Kinesiophobia (TSK-11) scores. Functional performance was assessed using single-leg hop distance (SLHD) and Limb Symmetry Index. In the operated knees, quadriceps (at 6 months postoperatively, *p* = 0.003) and hamstring (at 6 and 12 months postoperatively, *p* < 0.001) strength were significantly higher in the combined ACLR and ALLR group than the isolated ACLR group. The TSK-11 (at 6 and 12 months postoperatively, *p* < 0.001) was significantly lower in the combined ACLR and ALLR group than the isolated ACLR group. SLHD was significantly higher in the combined ACLR and ALLR group than the isolated ACLR group (at 6 months, *p* = 0.022 and at 12 months, *p* = 0.024). The addition of ALLR to primary ACLR yielded better muscle performance, fear of movement, and functional performance than isolated ACLR.

## Introduction

The anterior cruciate ligament (ACL) is commonly injured during sports activities, and ACL reconstruction (ACLR) is the current standard surgical treatment to regain knee joint stability and improve knee function^1–4^. Despite remarkably improved clinical outcomes following ACLR^5^, several recent studies reported that some patients still have difficulty returning to pre-injury sports participation levels after isolated ACLR^6–10^, possibly due to rotational knee instability at high levels of activity^11^. The addition of anterolateral ligament reconstruction (ALLR) to ACLR recently emerged as a potential solution^11–14^.

ALLR has attracted considerable attention for managing residual rotational instability since 2013^12,15,16^, and considerable research has investigated the anatomy and biomechanics of the anterolateral ligament (ALL). Moreover, an increasing number of studies have reported good clinical outcomes, including the pivot-shift test, graft failure rate, and patient-reported outcomes following ALLR combined with ACLR^5^. Additionally, a recent meta-analysis concluded that the addition of ALLR to ACLR could improve anteroposterior and anterolateral rotational stability of the knee joint and reduce the risk of failure^17^. However, there is still controversy regarding functional outcomes, such as knee muscle strength, between combined ACLR and ALLR and isolated ACLR^18,19^. Getgood et al.^18^ reported reduced quadriceps strength in the combined ACLR and ALLR at 6 months postoperative. However, Gillet et al.^19^ reported no intergroup differences in quadriceps or hamstring strength at 6 months postoperative. Therefore, to our knowledge, few studies have compared the functional outcomes of combined ACLR and ALLR versus isolated ACLR over time, and it remains unclear whether the addition of ALLR to ACLR could benefit functional outcomes such as muscle performance, proprioception, patient-reported outcomes, and functional performance. The rate of return to pre-injury sports activity levels after isolated ACLR is relatively low^20^. Considering that ACLR aims to restore structural stability and return patients to pre-injury sports activity levels, functional recovery outcomes should not be overlooked.

Therefore, this study aimed to compare the functional outcomes of combined ACLR and ALLR with those of isolated ACLR at four different time points (preoperatively and 3, 6, and 12 months postoperatively) up to 1 year following surgery. We hypothesized that the functional outcomes would be superior with combined ACLR and ALLR versus isolated ACLR.

## Methods

### Study design and participants

This prospective comparative study was approved by our local institutional review board (2018AN0261). A total of 250 patients who underwent primary ACLR using hamstring tendon autografts (semitendinosus and gracilis tendons) between July 2018 and October 2021 were enrolled. All participants provided written informed consent before participating. Overall, 200 patients were excluded from the study for the following reasons: bilateral ACL injury; revisional ACLR; other concomitant intra- or extra-articular injuries (i.e., meniscus, ligament, or ankle injuries); knee osteoarthritis (Kellgren–Lawrence grade > 1); vestibular or visual impairment; incomplete medical data or loss to follow-up; neurological pathology such as discogenic pain; and ACLR using allografts. The final analysis was performed based on data obtained from 50 patients (combined ACLR and ALLR in 24, isolated ACLR in 26) at four different time points (preoperative and 3, 6, and 12 months postoperative).

### Surgical technique

After anesthesia was administered and an aseptic dressing was positioned at the surgical site, routine arthroscopic examinations were performed using standard portals. Proper arthroscopic procedures were performed according to the intra-articular pathologies. Upon ACL rupture confirmation, a small skin incision for hamstring tendon harvest was made at the tibial tuberosity level on the anteromedial aspect of the proximal tibia. After meticulous soft-tissue dissection, the semitendinosus and gracilis tendons were harvested using a tendon stripper. A femoral tunnel was created on the anatomical femoral footprint using a FlipCutter drill (Arthrex, Naples, FL, USA) using the outside-in technique, while a tibial tunnel was created on the anatomical tibial footprint using an angled tibial guide. The prepared semitendinosus tendon graft was then inserted from the tibial tunnel to the femoral tunnel and fixed at the femoral and tibial sites using a cortical suspensory device and an interference screw with a post-tie method, respectively. If the patient desired an allograft, a tibialis allograft was used.

It was considered an indication for ALLR procedure if it included one or more of the following criteria: (1) chronic ACL tear, (2) pivot-shift ≥ grade 3, (3) high-level of sports activities such as pivot sports (soccer and basketball, etc.)^21^. In cases of combined ACLR and ALLR, ALLR was performed following ACLR using a gracilis tendon graft. A small skin incision was made in the lateral epicondyle area and a femoral tunnel was created 5 mm proximal and posterior to the lateral femoral epicondyle after an iliotibial band dissection. Two tibial bony sockets were created approximately 1 cm below the lateral joint line; one was located halfway between Gerdy’s tubercle and the tip of the fibular head, while the other was created approximately 10 mm anteriorly. The femoral side of the ALL graft was fixed using an interference screw, while the tibial side was fixed using a SwiveLock anchor screw (Arthrex, Naples, FL, USA).

### Rehabilitation protocol

All patients underwent the same postoperative rehabilitation protocol, which consisted of four phases. The participants visited our sports medical center once or twice a week for 12 weeks. The first phase (0–6 weeks postoperative) included the initial general recovery period. The second phase (6–12 weeks postoperative) was the next general function recovery. The third phase (13–24 weeks postoperative) was the functional performance recovery period. The final phase (24+ weeks postoperative) was the preparatory period for returning to sports activities including sport-specific technical training. A detailed rehabilitation protocol is provided in the Appendix.

### Outcome measures

#### Muscle performance

Muscle performance, proprioception, patient-reported outcomes, and functional performance were analyzed to compare functional outcomes between the two groups. The muscle performance test assessed muscle strength and acceleration time (AT)^22^. Quadriceps and hamstrings strength, as well as AT, were assessed using an isokinetic device (Biodex Multi-Joint System 4; Biodex Medical Systems, Inc. Shirly, NY, USA). Prior to the test session, each patient completed five warm-up repetitions of knee flexion and extension exercises. The testing order for limbs began with the uninvolved side. Patients performed up to 15 extension and flexion repetitions (concentric/concentric action mode) for each leg at 180°/sec while in an upright sitting position, with the maximal torque value recorded (Nm/kg)^23,24^. Flexor and extensor strengths were determined as the quadriceps and hamstring muscle strengths, respectively. AT represented the time taken to reach a preset angular velocity (180°/s in this study) during maximal knee flexion and extension, indicating the acceleration ability of the muscle. Hence, a fast AT was indicated greater muscle activation ability.

#### Proprioception

The reproduction of passive positioning (RPP) test was performed to assess joint position sense for knee joint proprioception^25^. The patients sat on an isokinetic chair with knees flexed at 90° and eyes closed. They were then asked to perform a predetermined knee extension (45° of knee flexion in this study) and hold it for 5 s with instructions to remember the position. The Biodex system moved the knee joint passively and the patients were asked to press a switch when the knee joint angle reached the target angle (45° of knee flexion). Differences between patient-instructed and target angles were recorded. The RPP test was performed twice on each leg with a 30-s rest period between tests. Positive values indicate that the angle instructed by the patient exceeded the target angle.

Dynamic postural stability was assessed using the overall Stability Index (OSI) using a Biodex Stability System (BSS; Biodex Medical Systems, Shirly, NY, USA)^26^. The foot platform of the BSS moved from 0° to 20° tilt with a 360° rotation. The level of stability automatically decreased by one level every 1.66 s from level 12 to 1 (most to least stable). The dynamic postural stability test was conducted with the participant standing barefoot on one leg. The patients completed two times, each for 20 s, with a 10-s rest period between them. A higher OSI indicated poorer dynamic postural stability.

#### Patient-reported outcomes

Patient-reported outcomes included the Lysholm Knee Scoring Scale, Tegner Activity Scale, International Knee Documentation Committee (IKDC), and Tampa Scale for Kinesiophobia (TSK-11) scores^27–29^. The Lysholm Knee Scoring Scale consisted of eight items: limping, pain, support, swelling, restraining, instability, squatting, and stair climbing. The IKDC score consisted of three items: symptoms, sports activities, and function. A lower IKDC and Lysholm Knee Scoring Scale score indicate more severe symptoms and poorer functional levels. The Tegner Activity Scale consisted of four items: activities of daily living, recreational sports, working, and competitive sports. A patient with a Tegner Activity Scale score ≥ 6 is presumed to participate in strenuous knee sports^30^. Lower scales indicate poorer knee function. The TSK-11 is a 11-item questionnaire used to evaluate the kinesiophobia or fear of movement, with high scores indicating a greater fear of movement, pain, and injury^31^. Based on previous studies, intra-class correlation coefficients for Lysholm^29^, Tegner^29^, IKDC^32^, TSK-11^33^ were 0.94, 0.82, 0.93, and 0.87, respectively.

#### Functional performance

Functional performance was evaluated using LSI and single-leg hop distance (SLHD). The LSI consisted of quadriceps muscle strength (LSI-quad) and hamstring muscle strength (LSI-hams). Based on previous study of the SLHD test^34,35^, the patients were instructed to poise themselves on one foot at the starting line, jump forward as far as possible, and land on the same foot. Landing with early touchdown of the contralateral foot or loss of balance was considered test failure. The average distances between the two trials were used in the analysis.

### Statistical analysis

The LSI value is the ratio of the muscle strength of the opposing limbs and calculated as the mean score for the injured limb divided by the uninjured limb × 100%^36^. A previous study demonstrated that the intergroup differences in quadriceps muscle strength (> 10%) were clinically significant after ACLR^37^. A priori power analysis was calculated using repeated-measures analysis of variance (RM-ANOVA), indicated that a minimum 48 patients (effect size *f*(V): 0.516, *P* (η^2^) = 0.210) would be needed to detect an intergroup difference in quadriceps muscle strength > 10% (α = 0.05, power = 0.8). In this study, the power for detecting a significant intergroup difference in quadriceps muscle strength was 0.830. Independent t-tests were used to examine continuous variables, while chi-square tests were used to examine categorical variables and compare demographic information between the two groups. Shapiro–Wilk's test and Levene's test were used to determine whether the normal distribution and assumption of equal variance were satisfied, respectively. For intersubject factors (isolated ACLR versus combined ACLR and ALLR) and intersubject factors (preoperative, 3, 6, and 12 months postoperative), RM-ANOVA was used to investigate group differences in outcomes by time point. If a significant interaction between time and group was found, a Bonferroni post hoc test was applied and corrected for *p* < 0.013. Partial eta squared (η^2^) was used to determine the effect size, with values of < 0.06 defined as small, 0.06 < x < 0.14 as medium, and > 0.14 as large (supplementary file)^38^. At the same time, correlations among SLHD, muscle strength, AT, RPP, OSI, patient-reported outcomes, and LSI were assessed by Pearson correlation analysis. For factors associated in the Pearson correlation analysis, a multiple linear regression analysis was performed to identify variables that independently affected SLHD in the operated knees. SLHD was defined as the dependent variable, while quadriceps muscle strength, hamstring muscle strength, and LSI-quad were defined as independent variables. The level of significance was set at *p* < 0.05.

### Ethical approval

Korea University Anam Hospital approved this study (2018AN0261). The study was performed in accordance with the ethical standards as laid out in the 1964 Declaration of Helsinki.

### Consent to participate

Informed consent was obtained from all individual participants included in the study.

## Results

No significant intergroup differences were found in age, weight, height, body mass index, concomitant meniscal injuries, time from injury to surgery, and sports activities (*p* > 0.05) (Table 1). Preoperatively, 25 (96.1%) patients in the isolated ACLR group and 24 (100%) patients in the combined ACLR and ALLR group had a grade 2 or 3 pivot shift (*p* > 0.05). At 12 months postoperatively, 13 (50%) patients in the isolated ACLR group and 18 (75%) patients in the combined ACLR and ALLR group showed a negative pivot shift (*p* = 0.026) (Table 1).

**Table 1:** Participants’ demographic data by study group.

	Combined ACLR and ALLR (n = 24)	Isolated ACLR (n = 26)	*P* value
Sex (male/female)	13/11	16/10	0.565
Age, years	29.4 ± 16.2	31.5 ± 13.5	0.095
Height, cm	174.2 ± 8.1	177.0 ± 7.5	0.898
Weight, kg	69.4 ± 10.6	71.1 ± 11.4	0.175
Body mass index, kg/m^2^	25.8 ± 4.3	27.1 ± 2.8	0.112
Injured side, right/left	19/5	20/6	1.0
Dominant knee, right/left	21/3	24/2	0.661
Time from injury to surgery, days	40.7 ± 31.2	29.7 ± 9.8	0.094
Sports and activity, n, low/high	7/17	9/17	0.767
Preoperative pivot shift grade (0/1/2/3), n	0/0/8/16	0/1/10/15	0.185
Postoperative 1-year pivot shift grade (0/1/2/3), n	18/6/0/0	13/10/3/0	0.026

Values are expressed as mean ± standard deviation or n as appropriate.

The pivot shift was graded as grade 0 (absent), grade 1 (glide), grade 2 (clunk), and grade 3 (gross).

ACLR, Anterior cruciate ligament reconstruction; ALLR, antero-lateral ligament reconstruction.

In the operated knees, a significant group effect was identified for the quadriceps strength with a medium effect size (*f* = 6.670, *p* = 0.013, η^2^ = 0.12), hamstring strength with a large effect size (*f* = 15.846, *p* < 0.001, η^2^ = 0.27) (Table S1), AT with a medium effect size (*f* = 6.181, *p* = 0.016, η^2^ = 0.14) (Table S2), and TSK-11 with a large effect size (*f* = 61.946, *p* < 0.001, η^2^ = 0.94) (Table S3).

**Figure 1:**
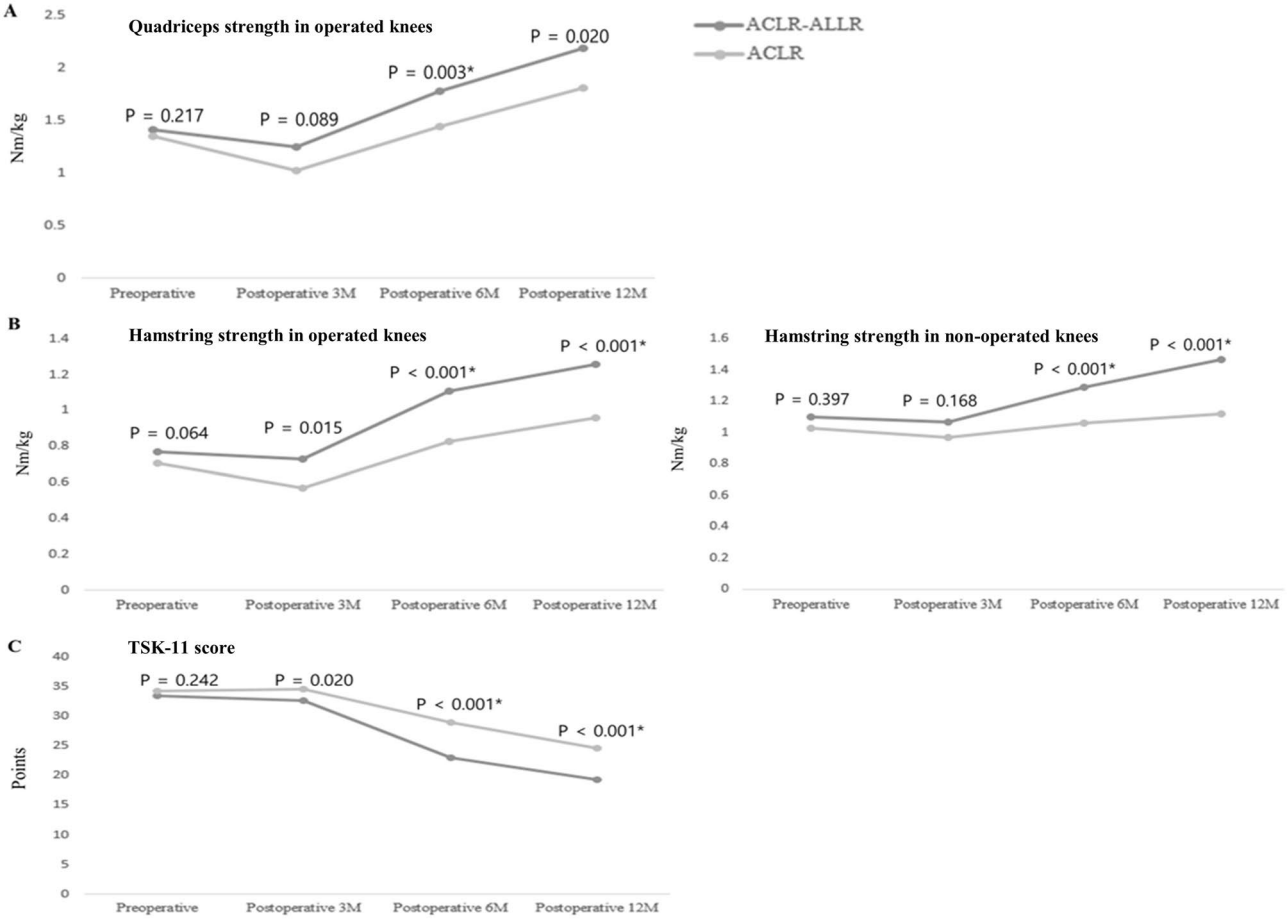
Comparison of functional outcomes between the two groups. (**A**) Quadriceps strength, (**B**) hamstring strength (left: operated knees, right: non-operated knees), (**C**) TSK-11 (kinesiophobia). *p < 0.013 compared between the two groups (post-hoc analysis).

Furthermore, significant group-by-time interactions were identified for quadriceps strength with a medium effect size (*f* = 2.896, *p* = 0.037, η^2^ = 0.06), for the hamstring strength with a medium effect size (*f* = 4.498, *p* = 0.006, η^2^ = 0.09) (Table S1), and for the TSK-11 with a large effect size (*f* = 30.029, *p* < 0.001, η^2^ = 0.39). However, no significant group-by-time effects were identified for other patient-reported outcomes such as Lysholm Knee Scoring Scale, IKDC, or Tegner Activity Scale scores (*p* > 0.05, Table S3). The subsequent independent t-test demonstrated that significant intergroup differences in quadriceps strength at 6 months postoperative (1.78 ± 0.5 vs. 1.44 ± 0.5, *p* = 0.003, Fig. 1), in hamstring strength at 6 and 12 months postoperative (1.11 ± 0.2 vs. 0.83 ± 0.2 and 1.26 ± 0.2 vs. 0.96 ± 0.2, *p* < 0.001, respectively, Fig. 1), and in the TSK-11 score at 6 and 12 months postoperative (23.0 ± 2.3 vs. 28.9 ± 1.8 and 19.4 ± 2.2 vs. 24.6 ± 1.9, *p* < 0.001, respectively, Fig. 1), indicating better quadriceps strength, hamstring strength, and TSK-11 scores for the combined ACLR and ALLR group.

A significant group-by-time interaction was found for the SLHD with a medium effect size (*f* = 5.583, *p* = 0.022, η^2^ = 0.10) (Table S4). The subsequent independent t-test demonstrated a significantly better SLHD at 6 and 12 months in the combined ACLR and ALLR group (101.6 ± 30.7 vs. 83.0 ± 28.4 and 115.5 ± 29.6 vs. 96.2 ± 32.6, *p* = 0.022 and *p* = 0.024, respectively). Among the parameters with significant intergroup differences, a correlation analysis of various parameters was performed for SLHD. A univariate analysis showed a significant correlation between quadriceps strength, hamstring strength, and LSI-quad. Multiple linear regression analysis of these three parameters showed that, at 6 months, LSI-quad (β = 0.633, *p* < 0.001) was a significant independent predictor for SLHD in the combined ACLR and ALLR group, whereas hamstring strength (β = 0.538, *p* = 0.001) and LSI-quad (β = 0.511, *p* = 0.001) were significant and independent predictor for SLHD in the isolated ACLR group. At 12 months, only LSI-quad was a significant independent predictor for SLHD in the combined ACLR and ALLR group (β = 0.425, *p* = 0.021) and isolated ACLR group (β = 0.513, *p* = 0.002).

In the non-operated knees, a significant group effect was found for quadriceps strength with a medium effect size (*f* = 5.131, *p* = 0.028, η^2^ = 0.10) and the hamstring strength with a large effect size (*f* = 8.392, *p* = 0.006, η^2^ = 0.15). In addition, significant group-by-time interactions were identified for hamstring strength with a medium effect size (*f* = 4.180, *p* < 0.010, η^2^ = 0.08) (Table S5). The subsequent independent t-test demonstrated significantly better hamstring strength in the combined ACLR and ALLR group at 6 and 12 months postoperative (1.29 ± 0.3 vs. 1.06 ± 0.3, *p* = 0.007 and 1.47 ± 0.3 vs. 1.12 ± 0.3, *p* < 0.001, respectively, Fig. 1). However, there were no significant intergroup differences in AT, RPP, or OSI (*p* > 0.05, Table S6).

## Discussion

This study aimed to compare the functional outcomes of combined ACLR and ALLR and isolated ACLR at four different time points (pre- and post-operative 3, 6, and 12 months). The most important finding in this study was that pivot shift, quadriceps strength, hamstring strength, TSK-11 score, AT, and SLHD were better in the operated knees of the combined ACLR and ALLR group than the isolated ACLR group at 6 and 12 months postoperative. Furthermore, SLHD was associated with LSI-quad in the combined ACLR and ALLR group at 6 months and with hamstring strength and LSI-quad in the isolated ACLR group, indicating that in the latter, the hamstrings might be an important factor in improving functional performance. Finally, in the non-operated knees, the quadriceps and hamstring strengths were better in the combined ACLR and ALLR group than in the isolated ACLR group.

Although there has been a recent increase in the number of studies reporting clinical outcomes following combined ACLR and ALLR, there is still a lack of studies comparing functional outcomes between combined ACLR and ALLR and isolated ACLR. In this study, better functional outcomes such as muscle strength, TSK-11 score, AT, and SLHD were identified in the combined ACLR and ALLR group compared with the isolated ALLR group at 6 and 12 months postoperatively. Considering that ACLR aims to restore structural stability and return patients to pre-injury sports levels, the recovery of functional outcomes is important. A recent meta-analysis demonstrated that combined ACLR and ALLR could improve anteroposterior and anterolateral rotational stability of the knee joint and reduce the risk of failure^17^. Pivot shift results were also better in the combined ACLR and ALLR group in this study. It is thought that better functional outcomes in the combined ACLR and ALLR group might be caused by enhanced postoperative rehabilitation through improved stability of the knee joint in this study.

In the present study, although knee muscle strength showed no significant differences between the two groups preoperatively and at 3 months postoperative, quadriceps strength at 6 months and hamstring strength at 6 and 12 months postoperative were significantly better in the combined ACLR and ALLR group compared with the isolated ACLR group. A possible explanation for this may be related to physical activity, such as postoperative Tegner Activity Scale scores. The Tegner Activity Scale is a reliable measure used to determine the level of physical activity and return to sports after an ACL injury^29,30,39^. In particular, patients with Tegner Activity Scale scores ≥ 6 can participate in recreational sports such as tennis, badminton, and handball^40^, which are associated with great knee muscle strength and self-efficacy^28,39,41^. In the present study, there were no statistical intergroup differences in Tegner Activity Scale scores at any time point (preoperative or 3, 6, and 12 months postoperative). However, the combined ACLR and ALLR group showed mean Tegner Activity Scale scores of 5.7 ± 1.0 at 6 months and 6.1 ± 1.1 at 12 months postoperative, whereas the isolated ACLR group showed mean Tegner Activity Scale scores of 4.8 ± 1.1 at 6 months and 5.4 ± 1.5 at 12 months. It is thought that improved muscle strength might be influenced by participation in strenuous knee sports. Beischer et al.^42^ reported that patients who successfully returned to sports after ACLR had higher self-efficacy and knee muscle strength than those who did not, indicating that high physical activity might be associated with better muscle function.

In the present study, although kinesiophobia (on the TSK-11) showed no significant difference preoperatively and at 3 months postoperative, it was significantly lower at 6 and 12 months postoperative (*p* < 0.001) in the combined ACLR and ALLR group compared with the isolated ACLR group. Recently, psychological factor is emphasized during rehabilitation after ACLR. Ohji et al. reported that kinesiophobia was moderately negatively associated with psychological readiness to return to sports^43^. After ACLR, patients who returned to knee-straining sports might have higher psychological readiness for return to sports than those who had not^39^. A recent systematic review and meta-analysis by Xiao et al.^44^ reported that after ACLR, kinesiophobia was lower in patients who returned to sports than in those who did not despite clinically similar patient-reported outcomes such as the IKDC scores. This may explain why patients who underwent combined ACLR and ALLR had better knee muscle performance and less kinesiophobia at 6 and 12 months postoperative than those who underwent isolated ACLR. A recent systematic review by Bakhsh et al.^45^ reported that insufficient muscle strength and inferior functional activities are associated with high-levels of kinesiophobia. Furthermore, it is possible that the lower muscle strength in the non-operated knees in the isolated ACLR versus combined ACLR and ALLR group was caused by lower physical activity levels. In addition to higher physical activity level, the thought of receiving additional ligament reinforcement could have provided a sense of psychological comfort to the patients.

In the present study, there was no difference in LSI for quadriceps and hamstring between the two group at 6 and 12 months. Our findings are consistent with the result of a previous study^46^. However, the combined ACLR and ALLR group showed better SLHD at 6 and 12 months postoperative than isolated ACLR group. This may be attributed to dynamic knee stability. The SLHD was used to evaluate dynamic knee stability^47^. A biomechanical study by Zee et al.^48^ found that tibial rotation increased during the SLHD test after isolated ACLR. Several systematic reviews and meta-analyses^17,49,50^ reported that the addition of ALLR to ACLR improves tibial rotational stability compared with isolated ACLR. In addition, as the muscle force generation capacity could improve dynamic joint stability, muscle performance, such as muscle strength and activation, could positively affect SLHD^23,47,51,52^. In the present study, muscle strength and AT for the quadriceps and hamstrings were better in the combined ACLR and ALLR versus isolated ACLR group. We also found that LSI-quad was a predictor of SLHD in the combined ACLR and ALLR group, whereas hamstring strength and LSI-quad were predictors of SLHD in the isolated ACLR group. These findings suggest that, enhanced LSI-quad and hamstring strength might improve SLHD by restoring dynamic knee stability in the combined ACLR and ALLR group and in isolated ACLR. However, to confirm that the LSI-quad and hamstring strength shown in our results represent predictor of SLHD in these patients, further evaluations should be done following rehabilitation.

This study has some limitations. Firstly, this study did not include a healthy control group. Secondly, no randomization was performed. Thirdly, while the number of patients exceeded the minimum calculated by the power analysis, the sample size remained relatively small. Therefore, further high-quality studies with larger participant cohorts, randomization, and longer follow-up durations are necessary to validate our findings. Fourthly, the rate of return to sport after ACLR was not measured, which could serve as a crucial indicator for comparing functional and clinical outcomes between the two groups. Finally, although functional performance was assessed using SLHD and LSI, these measures alone may be insufficient for evaluating the overall functional performance of patients post-ACLR.

### Clinical implication

At the 6 months, when specific training for a return to sports begins^53^, the addition of ALLR to ACLR may benefit muscle performance, fear of movement and readiness for return to activity/sport, and functional performance compared with isolated ACLR. These results suggest that additional ALLR should be considered to improve knee function, stability, and fear of movement in athletes or occupations requiring more dynamic knee stability.

## Conclusions

The addition of ALLR to primary ACLR resulted in better muscle performance, fear of movement, and functional performance than isolated ACLR up to 1 year postoperative.

### Supplementary Information

Supplementary Information 1.

Supplementary Tables.

## Data Availability

The data that support the findings of this study are available from author, Jin Hyuck Lee but restrictions apply to the availability of these data, which were used under license for the current study, and so are not publicly available. Furthermore, all data generated or analyzed during the current study will not be disclosed due to policy of the Korea University Anam Hospital Research Ethics Board.
