# Supplementary material for: Addition of anterolateral ligament reconstruction to primary anterior cruciate ligament reconstruction could benefit recovery of functional outcomes
Source: Sci Rep. 2024 May 20;14:11440. doi: 10.1038/s41598-024-62444-x (PMC11106076; doi:10.1038/s41598-024-62444-x)
Supplement: Supplementary file 1 — Supplementary Information 1. [file 41598_2024_62444_MOESM1_ESM.docx]

**Department of Sports Medical Center, Anam Hospital,**

**Korea University College of Medicine**

**ACL Reconstruction Rehabilitation Protocol**

**Phase 1. Immediately post-operatively to week 6**

Goals: The recovery period following the surgery

- Pain controlling, reducing swelling, recovering the range of motion, and normalizing gait pattern
- Control inflammation, swelling and pain
- Quadriceps activation
- Initial quadriceps/hamstring strengthening (without a lag)
- Active and passive knee full ROM
- Exercises for core, hip and calf muscles
- Education for walking with crutches & Restore normal gait on stable (level) surface

Exercises:

- Modalities ((ice, transcutaneous electrical nerve stimulation (TENS), interferential current therapy (ICT), ultrasound (US)
- Patella mobilization
- Quad sets
- Self passive/active ROM (wall slides, heels slides, heel prop, extension overpressure)
- Ankle pump
- PWB-> FWB gait training (with and without crutches and brace)
- Straight leg raises with neuromuscular electrical stimulation (NMES)
- Terminal knee extension (CKC -> OKC, consider NMES)
- Calf/Hamstring stretching and strengthening
- 4-direction (flexion, extension, adduction, abduction) leg strengthening in full extension with elastic band
- Weight shifting and bearing (single leg balance)
- Isometric wall squat (limit to 90°)
- Leg press (limited ROM, tolerable, starting at 3 weeks)
- Sitting knee extension (isometric -> band exercise, starting at 4 weeks)

**Phase 2. Post-operatively weeks 6 to 12**

Goals: The recovery period of general function

- Muscle strengthening & proprioception training
- Continue flexibility and ROM
- Progressive thigh, hip, calf and core strengthening
- Increase motor control/proprioception
- Increase muscle strengthening
- Restore normal stair-climbing

Exercises:

- Progressive balance training (stable to unstable surface/both to single)
- Hip strengthening (clam shell, hip bridge, hip abduction and etc.)
- Core training (forward plank, side plank, dead burg and etc.)
- Stationary bike
- Hamstring stretches
- CKC training (squat and lunge, leg press, step ups/downs, bridges and etc.)
- OKC training (leg extension/curl with elastic band -> machine leg extension/curl)
- Isokinetic machine training (high angular velocity)- begins at 10 weeks

**Phase 3. Post-operatively weeks 13 to 24**

Goals: The recovery period of functional performance

- Enhance muscle strength, proprioception, and neuromuscular control (plyometric and agility)
- Achieved approximately 90% LSI scores (Isokinetic test at 24 weeks)
- Achieved symmetric balance scores (machine balance test at 24 weeks)
- Achieved above 85% hop test scores (functional test at 24 weeks)
- Restore normal running mechanics
- Increase quadriceps/hamstring strengthening
- Increase variability

Exercises:

- Transition to gym-based program and/or supervised training
- Cardiovascular training (bike and running)
- Advance motor control training
- High intensity strength training
- Plyometric training
- Progressive running training (distance)
- Progressive motor control training
- Agilities training (side steps, ladder drills, one or double jump and etc)

**Phase 4. Post-operatively weeks 24~**

Goals: The preparation period for returning to sports

- Sport-specific technical training
- Achieved above 90% LSI scores (continue Isokinetic test at 9 and 12 months)
- Achieved symmetric balance scores (continue machine balance test at 9 and 12 months)
- Achieved above 90% hop test scores (continue functional performance test at 9 and 12 months)
- Safe return to sports/work
- Restoration of conditioning level to pre-injury level
- Sport specific skill acquisition

Exercises:

- High intensity power training and maximal effort strength training
- Progressive agilities and motor control training
- Specific training for individualized to sport/position
- Check strength, postural stability and functional performance test (at 9 and 12 months)
- Education for secondary prevention
