# Supplementary material for: Addition of anterolateral ligament reconstruction to primary anterior cruciate ligament reconstruction could benefit recovery of functional outcomes
Source: Sci Rep. 2024 May 20;14:11440. doi: 10.1038/s41598-024-62444-x (PMC11106076; doi:10.1038/s41598-024-62444-x)
Supplement: Supplementary file 2 — Supplementary Tables. [file 41598_2024_62444_MOESM2_ESM.docx]

**Table S1.** Comparative muscle strength outcomes of operated knees of the ACLR and ALLR versus isolated ACLR groups

|  | | Combined ACLR and ALLR | Isolated ACLR |  |  |  |  |
| --- | --- | --- | --- | --- | --- | --- | --- |
| Variable (operated side) | Time | Mean±SD | Mean±SD | Source | F | P^1^ (η^2^) | P^2^ (post-hoc analysis) |
| Quadriceps strength (Nm/kg) | Pre | 1.41±0.4 | 1.35±0.5 | Group  Time  Group × Time | 6.670  55.940  2.896 | 0.013 (0.12)  <0.001 (0.54)  0.037 (0.06) | 0.217 |
|  | Post 3M | 1.25±0.5 | 1.02±0.5 |  |  |  | 0.089 |
|  | Post 6M | 1.78±0.5 | 1.44±0.5 |  |  |  | **0.003 ^*^** |
|  | Post 1Y | 2.19±0.6 | 1.81±0.5 |  |  |  | 0.020 |
| Hamstring strength (Nm/kg) | Pre | 0.77±0.2 | 0.71±0.3 | Group  Time  Group × Time | 15.846  66.006  4.498 | <0.001 (0.27)  <0.001 (0.52)  0.006 (0.09) | 0.064 |
|  | Post 3M | 0.73±0.2 | 0.57±0.2 |  |  |  | 0.015 |
|  | Post 6M | 1.11±0.2 | 0.83±0.2 |  |  |  | **<0.001 ^*^** |
|  | Post 1Y | 1.26±0.2 | 0.96±0.2 |  |  |  | **<0.001 ^*^** |

1Y, 1 year; 3M, 3 months; 6M, 6 months; ACLR, anterior cruciate ligament reconstruction; ALLR, anterolateral ligament reconstruction; H/Q, hamstring-to-quadriceps muscle ratio; Pre, preoperative; Post, postoperative; SD, standard deviation
P^1^: p-value of RM-ANOVA, p < 0.05
P^2^: p-value of post-hoc analysis, *p < 0.013

**Table S2.** Comparative muscle acceleration time and proprioception outcomes of operated knees in the combined ACLR and ALLR and isolated ACLR groups

|  | | Combined ACLR and ALLR | Isolated ACLR |  |  |  |  |
| --- | --- | --- | --- | --- | --- | --- | --- |
| Variable (operated side) | Time | Mean±SD | Mean±SD | Source | F | P^1^ (η^2^) | P^2^ (post-hoc analysis) |
| Quadriceps AT (ms) | Pre | 67.1±24.6 | 76.2±31.8 | Group  Time  Group × Time | 6.181  7.860  0.452 | 0.016 (0.11)  0.001 (0.14)  0.672 (0.01) | **^a^** |
|  | Post 3M | 66.7±25.1 | 81.5±37.9 |  |  |  |  |
|  | Post 6M | 54.2±11.4 | 72.8±25.2 |  |  |  |  |
|  | Post 1Y | 52.1±14.7 | 58.5±15.9 |  |  |  |  |
| Hamstring AT (ms) | Pre | 87.5±26.9 | 97.3±47.3 | Group  Time  Group × Time | 3.132  3.438  0.564 | 0.083 (0.06)  0.025 (0.07)  0.612 (0.01) | **^a^** |
|  | Post 3M | 80.4±21.2 | 97.3±39.9 |  |  |  |  |
|  | Post 6M | 75.8±17.2 | 80.0±18.3 |  |  |  |  |
|  | Post 1Y | 77.9±16.7 | 86.5±27.6 |  |  |  |  |
| RPP (°) | Pre | -3.5±7.1 | -3.8±9.3 | Group  Time  Group × Time | 1.300  3.868  0.592 | 0.260 (0.03)  0.018 (0.08)  0.583 (0.01) | **^a^** |
|  | Post 3M | -1.1±4.5 | -3.8±8.7 |  |  |  |  |
|  | Post 6M | -0.2±2.4 | -3.2±5.9 |  |  |  |  |
|  | Post 1Y | -0.2±1.8 | -0.8±4.9 |  |  |  |  |
| OSI (°) | Pre | 2.9±1.4 | 2.8±1.1 | Group  Time  Group × Time | 0.464  5.487  0.479 | 0.499 (0.01)  0.002 (0.10)  0.685 (0.01) | **^a^** |
|  | Post 3M | 2.9±1.5 | 3.0±1.4 |  |  |  |  |
|  | Post 6M | 2.5±0.8 | 2.7±1.0 |  |  |  |  |
|  | Post 1Y | 2.2±0.8 | 2.5±1.1 |  |  |  |  |

1Y, 1 year; 3M, 3 months; 6M, 6 months; ACLR, anterior cruciate ligament reconstruction; ALLR, anterolateral ligament reconstruction; AT, acceleration time; OSI, Overall Stability Index; Post, postoperative; Pre, preoperative; RPP, reproduction of passive positioning; SD, standard deviation

P^1^: p-value of RM-ANOVA, p < 0.05
P^2^: p-value of post-hoc analysis, p < 0.013

**^a^** Post-hoc analysis was not performed because no significant interaction between time and group was found in RM-ANOVA (P^1^ > 0.05).

**Table S3.** Comparative patient-reported outcomes by study group

|  | | Combined ACLR and ALLR | Isolated ACLR |  |  |  |  |
| --- | --- | --- | --- | --- | --- | --- | --- |
| Variable | Time | Mean±SD | Mean±SD | Source | F | P^1^ (η^2^) | P^2^ (post-hoc analysis) |
| Lysholm (%) | Pre | 63.4±20.6 | 61.4±18.9 | Group  Time  Group × Time | 1.157  16.937  0.203 | 0.288 (0.02)  0.001 (0.26)  0.847 (0.01) | **^a^** |
|  | Post 3M | 67.9±15.3 | 65.5±20.4 |  |  |  |  |
|  | Post 6M | 77.7±12.2 | 72.5±15.4 |  |  |  |  |
|  | Post 1Y | 81.5±14.3 | 76.5±14.0 |  |  |  |  |
| Tegner (p) | Pre | 7.0±0.9 | 7.1±1.0 | Group  Time  Group × Time | 3.575  150.178  1.552 | 0.065 (0.07)  <0.001 (0.76)  4.087 (0.08) | **^a^** |
|  | Post 3M | 3.5±1.1 | 3.3±1.0 |  |  |  |  |
|  | Post 6M | 5.7±1.0 | 4.8±1.1 |  |  |  |  |
|  | Post 1Y | 6.1±1.1 | 5.4 ± 1.5 |  |  |  |  |
| IKDC (%) | Pre | 53.4±17.4 | 53.6±19.9 | Group  Time  Group × Time | 0.701  38.958  0.357 | 0.407 (0.01)  0.001 (0.45)  0.702 (0.01) | **^a^** |
|  | Post 3M | 59.7±13.9 | 55.8±14.0 |  |  |  |  |
|  | Post 6M | 72.9±12.5 | 70.2±15.5 |  |  |  |  |
|  | Post 1Y | 77.7±9.7 | 73.3±17.5 |  |  |  |  |
| TSK-11(p) | Pre | 33.5±2.6 | 34.3±2.7 | Group  Time  Group × Time | 61.946  788.424  30.029 | <0.001 (0.94)  <0.001 (0.99)  <0.001 (0.39) | 0.242 |
|  | Post 3M | 32.7±3.1 | 34.5±2.2 |  |  |  | 0.020 |
|  | Post 6M | 23.0±2.3 | 28.9±1.8 |  |  |  | **<0.001^*^** |
|  | Post 1Y | 19.4±2.2 | 24.6±1.9 |  |  |  | **<0.001 ^*^** |

1Y, 1 year; 3M, 3 months; 6M, 6 months; ACLR, anterior cruciate ligament reconstruction; ALLR, anterolateral ligament reconstruction; IKDC, International Knee Documentation Committee; Post, postoperative; Pre, preoperative; SD, standard deviation; TSK, Tampa Scale for Kinesiophobia

Patient-reported outcomes are shown as percentages (%) or points (p).

P^1^: p-value of RM-ANOVA, p < 0.05
P^2^: p-value of post-hoc analysis, p < 0.013

**^a^** Post-hoc analysis was not performed because no significant interaction between time and group was found in RM-ANOVA (P^1^ > 0.05).

**Table S4.** Comparative LSI and SLHD by study group using repeated-measures analysis of variance

|  | | Combined ACLR and ALLR | Isolated ACLR |  |  |  |  |
| --- | --- | --- | --- | --- | --- | --- | --- |
| Variable (operated side) | Time | Mean±SD | Mean±SD | Source | F | P^1^ (η^2^) | P^2^ (post-hoc analysis) |
| LSI-quadriceps (%) | Pre | 61.3±17.9 | 61.7±21.2 | Group  Time  Group × Time | 3.541  10.361  1.393 | 0.066 (0.07)  0.002 (0.18)  0.244 (0.03) | **^a^** |
|  | Post 3M | 53.7±20.4 | 51.4±18.8 |  |  |  |  |
|  | Post 6M | 78.1±14.8 | 73.2±20.5 |  |  |  |  |
|  | Post 1Y | 89.6±18.1 | 78.5±16.2 |  |  |  |  |
| LSI-hamstring (%) | Pre | 70.1±16.2 | 69.3±14.8 | Group  Time  Group × Time | 1.136  3.681  3.446 | 0.292 (0.02)  0.061 (0.07)  0.070 (0.07) | **^a^** |
|  | Post 3M | 68.5±16.7 | 59.2±13.4 |  |  |  |  |
|  | Post 6M | 87.1±12.8 | 79.6±15.7 |  |  |  |  |
|  | Post 1Y | 88.2±12.8 | 87.2±15.0 |  |  |  |  |
| Single-leg hop distance (cm) | Pre | - | - | Group  Time  Group × Time | 5.583  19.117  0.014 | 0.022 (0.10)  <0.001 (0.29)  0.905 (0.01) | **^a^** |
|  | Post 3M | - | - |  |  |  |  |
|  | Post 6M | 101.6±30.7 | 83.0±28.4 |  |  |  | **0.022^†^** |
|  | Post 1Y | 115.5±29.6 | 96.2±32.6 |  |  |  | **0.024^†^** |

1Y, 1 year; 3M, 3 months; 6M, 6 months; ACLR, anterior cruciate ligament reconstruction; ALLR, anterolateral ligament reconstruction; LSI, Limb Symmetry Index; SD, standard deviation; SLHD, single-leg hop distance

P^1^: p-value of RM-ANOVA, p < 0.05
P^2^: p-value of post-hoc analysis, **^†^**p <0.025

**^a^** Post-hoc analysis was not performed because no significant interaction between time and group was found in RM-ANOVA (P^1^ > 0.05).

**Table S5.** Comparative muscle strength outcomes of non-operated knees by study group

|  | | Combined ACLR and ALLR | Isolated ACLR |  |  |  |  |
| --- | --- | --- | --- | --- | --- | --- | --- |
| Variable (non-operated side) | Time | Mean±SD | Mean±SD | Source | F | P^1^ (η^2^) | P^2^ (post-hoc analysis) |
| Quadriceps strength (Nm/kg) | Pre | 2.30±0.6 | 2.18±0.3 | Group  Time  Group × Time | 5.131  9.003  1.348 | 0.028 (0.10)  <0.001 (0.16)  0.262 (0.03) | **^a^** |
|  | Post 3M | 2.32±0.5 | 1.98±0.5 |  |  |  |  |
|  | Post 6M | 2.51±0.5 | 2.21±0.5 |  |  |  |  |
|  | Post 1Y | 2.62±0.6 | 2.29±0.5 |  |  |  |  |
| Hamstring strength (Nm/kg) | Pre | 1.10±0.3 | 1.03±0.3 | Group  Time  Group × Time | 8.392  16.976  4.180 | 0.006 (0.15)  <0.001 (0.26)  0.010 (0.08) | 0.397 |
|  | Post 3M | 1.07±0.3 | 0.97±0.3 |  |  |  | 0.168 |
|  | Post 6M | 1.29±0.3 | 1.06±0.3 |  |  |  | **<0.001 ^*^** |
|  | Post 1Y | 1.47±0.3 | 1.12±0.3 |  |  |  | **<0.001 ^*^** |

1Y, 1 year; 3M, 3 months; 6M, 6 months; ACLR, anterior cruciate ligament reconstruction; ALLR, anterolateral ligament reconstruction; H/Q, hamstring-to-quadriceps muscle ratio; Post, postoperative; Pre, preoperative; SD, standard deviation

P^1^: p-value of RM-ANOVA, p < 0.05
P^2^: p-value of post-hoc analysis, *p < 0.013

**^a^** Post-hoc analysis was not performed because no significant interaction between time and group was found in RM-ANOVA (P^1^ > 0.05).

**Table S6.** Comparative muscle acceleration time and proprioception outcomes of non-operated knees by study group

|  | | Combined ACLR and ALLR | Isolated ACLR |  |  |  |  |
| --- | --- | --- | --- | --- | --- | --- | --- |
| Variable (non-operated side) | Time | Mean±SD | Mean±SD | Source | F | P^1^ (η^2^) | P^2^ (post-hoc analysis) |
| Quadriceps AT (ms) | Pre | 57.5±17.9 | 60.8±16.5 | Group  Time  Group × Time | 1.868  3.538  0.201 | 0.178 (0.04)  0.023 (0.07)  0.865 (0.01) | **^a^** |
|  | Post 3M | 50.8±17.2 | 57.7±23.7 |  |  |  |  |
|  | Post 6M | 47.9±10.6 | 51.9±16.3 |  |  |  |  |
|  | Post 1Y | 50.0 ± 11.8 | 52.3 ± 18.4 |  |  |  |  |
| Hamstring AT (ms) | Pre | 85.0±26.5 | 81.5±28.5 | Group  Time  Group × Time | 0.147  0.795  0.627 | 0.703 (0.01)  0.493 (0.02)  0.590 (0.01) | **^a^** |
|  | Post 3M | 78.8±28.0 | 80.4±29.5 |  |  |  |  |
|  | Post 6M | 76.7±19.9 | 78.5±19.3 |  |  |  |  |
|  | Post 1Y | 75.0±10.2 | 82.3±21.9 |  |  |  |  |
| RPP (°) | Pre | -1.7±6.1 | 0.9±7.3 | Group  Time  Group × Time | 0.697  0.291  0.877 | 0.408 (0.01)  0.291 (0.03)  0.448 (0.02) | **^a^** |
|  | Post 3M | -1.7±3.8 | -1.3±7.7 |  |  |  |  |
|  | Post 6M | -0.3±2.9 | -0.3±5.5 |  |  |  |  |
|  | Post 1Y | -0.3±2.4 | 0.4±3.3 |  |  |  |  |
| OSI (°) | Pre | 2.8±1.5 | 2.7±1.0 | Group  Time  Group × Time | 0.016  4.880  1.042 | 0.899 (0.01)  0.004 (0.09)  0.372 (0.02) | **^a^** |
|  | Post 3M | 2.7±1.3 | 2.9±1.1 |  |  |  |  |
|  | Post 6M | 2.7±1.2 | 2.5±1.3 |  |  |  |  |
|  | Post 1Y | 2.1±0.9 | 2.4±0.9 |  |  |  |  |

1Y, 1 year; 3M, 3 months; 6M, 6 months; ACLR, anterior cruciate ligament reconstruction; ALLR, anterolateral ligament reconstruction; AT, acceleration time; Post, postoperative; Pre, preoperative; RPP, reproduction of passive positioning; OSI, Overall Stability Index; SD, standard deviation
P^1^: p-value of RM-ANOVA, p < 0.05
P^2^: p-value of post-hoc analysis, p < 0.013

**^a^** Post-hoc analysis was not performed because no significant interaction between time and group was found in RM-ANOVA (P^1^ > 0.05).
